# Rotating Minimal Thermodynamic Systems

**DOI:** 10.3390/e24020168

**Published:** 2022-01-23

**Authors:** Edward Bormashenko

**Affiliations:** Chemical Engineering Department, Engineering Sciences Faculty, Ariel University, Ariel 407000, Israel; edward@ariel.ac.il

**Keywords:** rotating systems, double-well potential, inertia force, minimal thermal engine, Landauer bound, friction, symmetry

## Abstract

Minimal rotating thermodynamic systems are addressed. Particle *m* placed into the rotating symmetrical double-well potential (bowl), providing binary logical system is considered. The condition providing the transfer of the particle from one frictionless half-well to another, and, in this way, the possibility to record 1 bit of information is derived. The procedure of recording turns out to be irreversible; it is impossible to return the particle to its initial state under rotation about the same axis. The same rotating double-well system exerted to the thermal noise is considered. A minimal rotating thermal engine built of the rotating chamber, movable partition, and the particle confined within the chamber is treated. Rotation of the system displaces the partition, thus enabling erasing of one bit information. Erasing of 1 bit of information is due to the inertia (centrifugal force) acting on the partition. Isothermal expansion of the “minimal gas” expectedly gives rise to the Landauer bound. Compression of the “gas” with the rotation around the same axis is impossible and demands the additional axis of rotation. The interrelation between the possibility of recording/erasing information and the symmetry of the system is considered.

## 1. Introduction

Explaining of the physical origin of “the arrow of time” remains one of the most important problems of the modern physics [1,2,3,4,5,6,7,8,9,10]. Despite our innate sense of time as unidirectional flow, the self-consistent physical point of view leads to the possibility that temporal passage (time arrow) is illusory [4]. It is well-known that every solution of a dynamical law is accompanied by a time-reversed solution, and initial conditions can always be reformulated as final conditions [5]. The laws of physics do not distinguish the future from the past direction of time [5]. No asymmetry is contained in the laws of mechanics (classical or quantum) and electrodynamics [5]. The CPT theorem states that the laws are invariant under the combination of charge conjugation, space inversion, and time reversal [2]. 

However, we are well-acquainted with the numerous irreversible physical, chemical, and biological processes, exemplifying what is called “the arrow of time”. The Second Law of Thermodynamics states, in particular, that the entropy of isolated systems left to spontaneous evolution cannot decrease, thus dictating the direction of flight of the arrow of time from past to future and predicting irreversibility of the processes occurring in macroscopic physical systems built of the large number of particles. The question is how these irreversible processes arise from the completely reversible physics laws. It sometimes suggested that the Second Law is not a true physical law but rather a consequence of the universe’s initial conditions; under special initial conditions, the Second law can be reversed. Albert Einstein once wrote: “People like us who believe in physics know that the distinction between past, present, and future is only a stubbornly persistent illusion”. It was demonstrated in [3] that the possibility to refute the Second Law of Thermodynamics implies to perfect determinism, pre-supposed in the physical system. Elitzur and Dolev demonstrated that with even the slightest failure of determinism, an intrinsic arrow of time must emerge in any closed system, regardless of its initial conditions, but with perfect accordance with the time arrow of the entire universe, despite the system’s isolation [3]. It was demonstrated by Riek that the discrete nature of time implies irreversibility of physical processes [7,8,9]. Gujrati suggested that the cause of stochasticity in a statistical approach arises from interactions with the surrounding medium. The stochasticity destroys temporal symmetry and homogeneity [10]. Ben-Naim, in turn, argued recently that that entropy is a timeless quantity; thus, the Second Law of Thermodynamic is not directly related to “the arrow of time” and the paradox emerges from confusing Shannon’s measure of information with the thermodynamic entropy [11]. 

In our paper, we discuss “the arrow of time” problem in the perspective of small thermodynamic systems [12,13] and the Landauer principle, establishing the connection between information and thermodynamics [14,15,16,17]. 

## 2. Results and Discussion

### 2.1. Particle Placed in a Double-Well Potential Exerted to the Centrifugal Force 

**Figure 1:**
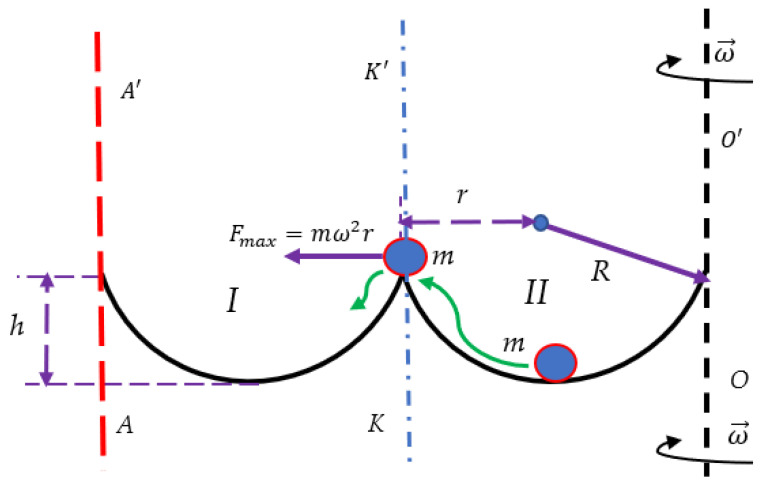
Particle m is placed into the twin-well, symmetrical, frictionless bowl, built of two identical spherical wells labeled “I” and “II”. The initial location of the particle is confined within Well “II”. Location of the particle in the well labeled “II” corresponds to the informational state “0”; the location of the particle in the Well “I” corresponds to the informational state “1”. The system is rotating with an angular velocity $\vec{\omega }\;$ around the vertical axis $OO^{\prime }.\;$ The inertial (centrifugal) force transfers the particle from the Well “II” to the Well “I” (the green arrow indicates the path of the particle, driven by the centrifugal force). Vertical axis $KK^{\prime }$ coincides with the axis of symmetry of the twin-well bowl.

Consider particle *m* placed into the symmetrical double-well potential, as shown in Figure 1. The particle can be stably trapped in either the left or right half-well, corresponding to informational states “0” and “1”, as suggested in the original papers by Rolph Landauer (see [14,15]). For the sake of simplicity, assume the particle is placed into the twin-well, symmetrical, frictionless bowl, built of two identical spherically shaped wells labeled “I” and “II”. Location of the particle in Well “II” corresponds to “0” state, whereas the particle trapped within Well “I” corresponds, in turn, to “1” state of our minimal computing device. The radius of the bowl is *R*, and its height is *h* (see Figure 1). Assume that initially, the particle *m* is in rest at the bottom of the potential Well “II”, as shown in Figure 1. Now let us rotate the entire system with an angular velocity $\vec{\omega }$ around vertical axis $OO^{\prime },$ as depicted in Figure 1. Now the particle is exerted to inertial (centrifugal) force. The maximal value of this force equals $F_{max}=m\omega^{2}r$ (see Figure 1). If the condition given by Equation (1) takes place:

(1) $$h<R-\frac{g}{\omega^{2}}$$

the particle will pass from Well “II” to Well “I”. Equation (1) is easily re-shaped as:

(2) $$\omega >\sqrt{\frac{g}{R-h}}$$

Obviously, the transfer of the particle becomes possible if the condition h<R is kept. Thus, it is seen, that the centrifugal force may record information in the addressed system, when Inequality 2 takes place. The suggested Gedanken Experiment resembles that reported in [18], in which the inertia force arising from the accelerated translational motion of the symmetrical double-well potential was exploited for recording/erasing of information. However, we demonstrate now that the inertia forces emerging from the rotation of the double-well bowl give rise to very different informational consequences. Now let us try to erase the recorded bit of information and return the particle to the potential Well “II”. Let us change the rotation of the bowl from $\vec{\omega }$ to $-\vec{\omega }$ (simply speaking change the clockwise rotation to the counter-clockwise). It is immediately recognized that such a switch of the rotation direction will not return the particle to Well “II”, it will push the particle from the Well “I”. 

**Figure 2:**
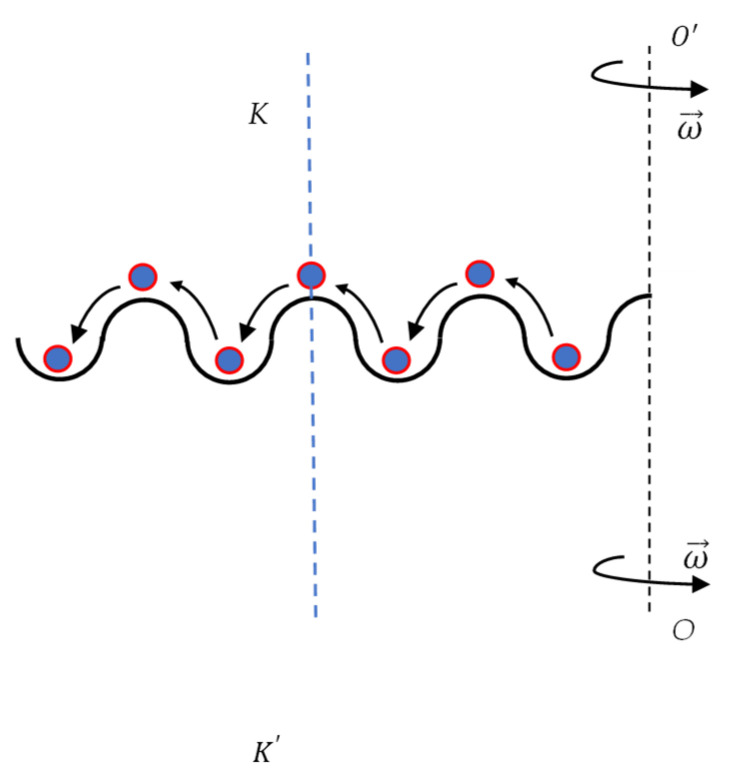
Inertia force enables recording of the information in the potential comb. The continuously increasing inertia force transfers the particle from well to well; thus, recording the information. Vertical axis $KK^{\prime }$ coincides with the vertical axis of symmetry of the potential comb.

Consider now particle *m* confined within the potential comb built of identical potential wells depicted in Figure 2. The continuous further transport of the particle along the potential comb becomes possible, which will record additional bits of information, as shown in Figure 2. The centrifugal force will be increased with distance from the axis; hence, it will inevitably transfer the particle along the comb to the adjacent well as illustrated in Figure 2, whatever the direction of the rotation. It should be emphasized that rotation across the vertical axis $\;OO^{\prime }$ does not enable erasing of information by reversion of the direction of rotation of the system; the rotation around the $OO^{\prime }$ axis will give rise to recording of information only. If we want to erase the information and to return the particle to the Well “I”, we have necessarily to change the axis of rotation and to exploit the vertical axis $AA^{\prime }\;$ depicted with the red dashed line in Figure 1. 

**Figure 3:**
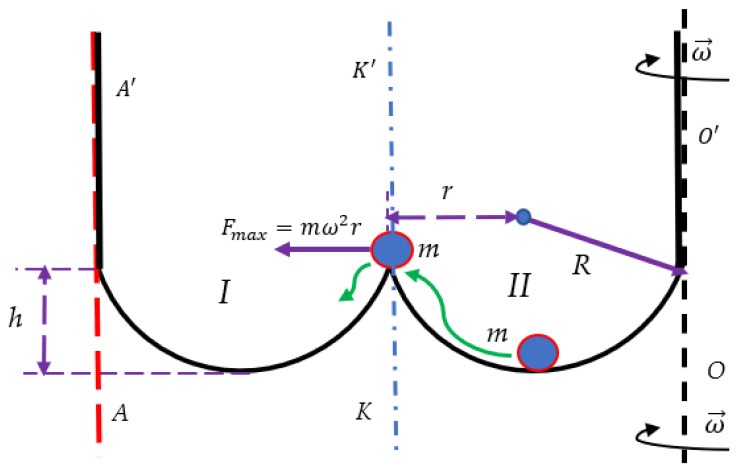
Particle m is placed into the twin-well, symmetrical, frictionless bowl, built of two identical spherical wells labeled “I” and “II”. The confining wells of the bowl are suggested to be infinitely high. The initial location of the particle is confined within Well “II”. The system is rotating with an angular velocity $\vec{\omega }$ around the vertical axis $OO^{\prime }.$ The inertial (centrifugal) force transfers the particle from the Well “II” to the Well “I” (the green arrow indicates the path of the particle, driven by the centrifugal force). The rotation will bring the particle to Well “I” (or leave within Well “I”), whatever the initial location of the particle. Vertical axis $KK^{\prime }$ coincides with the axis of symmetry of the twin-well bowl.

Consider now the particle *m* confined within the potential well depicted in Figure 3. The confining walls of the well are supposed to be infinitely high. Initially, the particle *m* is located at the bottom of the potential Well “II”. In this case, the rotation of the entire system around vertical axis $OO^{\prime }$ with an angular velocity $\vec{\omega }$ ($\omega >\sqrt{\frac{g}{R-h}}$) will transfer the particle from Well “II” to Well “I”, in which the particle will be trapped. If the particle was located initially in the Well “I”, the same rotation will leave the particle in the Well “I”. Thus, the aforementioned rotation will bring/leave the particle to/within Well “I”, whatever the initial location of the particle. 

Until now, we have completely neglected the friction or dissipation processes. We latently assumed that the transfer of the particle from the Well “II” to Well “I” is frictionless. Now, consider the dissipation processes (or alternatively thermal noise). If we assume that our particle *m* is in a thermal equilibrium with isothermal surrounding *T* the thermal noise ca, $k_{B}T$ ($k_{B}$ is the Boltzmann constant) should be surmounted in order to transfer the particle from the ZERO state to the ONE state. This reasoning leads to the obvious condition:

(3) $$\;\;\;m\omega^{2}r^{2}\geq k_{B}T$$

Considering $r^{2}=2hR-h^{2}$ yields:

(4) $$\omega \geq \sqrt{\frac{k_{B}T}{m\left(2hR-h^{2}\right)}}$$

Combining Equations (2) and (4) gives rise to Equation (5), representing the condition necessary for recording of 1 bit of information in the addressed system considering thermal noise:

(5) $$\omega \geq max\left\{\sqrt{\frac{k_{B}T}{m\left(2hR-h^{2}\right)}};\;\sqrt{\frac{g}{R-h}}\right\}$$

We conclude that that single-axis double-well system rotating system depicted in Figure 1 enables irreversible recording of information if Equation (5) takes place. Actually, rotation about the $OO^{\prime }$ axis breaks the symmetry of the double-well system; the direction of the inertia force acting on the particle does not switch under the change of the direction of rotation of the entire system. This symmetry breaking results in the irreversibility of the recording of information, indicating the emerging “arrow of time” in the addressed system. Note that rotation of the system around vertical axis $KK^{\prime }$ coinciding with the axis of symmetry of the twin-well bowl does not allow transfer of the particle from Well “II” to Well “I” (see Figure 1 and Figure 3); thus, irreversible recording of information in the suggested system becomes impossible for both wells depicted in Figure 1 and Figure 3. Consider now rotation of the potential comb depicted in Figure 2 around axis $KK^{\prime }$ coinciding with the vertical axis of symmetry of the comb. Placing of the particle *m* into one of the wells immediately breaks the symmetry of the entire system (namely: potential comb + particle *m*), enabling recording of information under rotation of the comb around axis $KK^{\prime }.$ The aforementioned considerations shed light on the non-obvious connection between the symmetry of the system and possibility to record information within its “memory units”. 

Now consider the friction-induced dissipation. It is noteworthy that the Stokes-like friction force f=−kv, where *k* is the friction coefficient and *v* is the velocity of the particle *m*, destabilizes the initial equilibrium of the particle in the h=0 point, and when $\omega^{2}>\frac{g}{R}$ takes place, the particle will be brought to Well “I” along the ever widening spiral path [19,20,21]. Thus, the Stokes-likes friction force promotes irreversible recording of information in the addressed twin-well system, depicted in Figure 1. It also should be mentioned that, starting from the second well of the potential ratchet, depicted in Figure 2, the destabilizing action of the friction force becomes unnecessary for the transfer of the particle along the ratchet; the continuously increasing centrifugal force will transfer the particle. 

### 2.2. Minimal Rotating Thermal Engine

**Figure 4:**
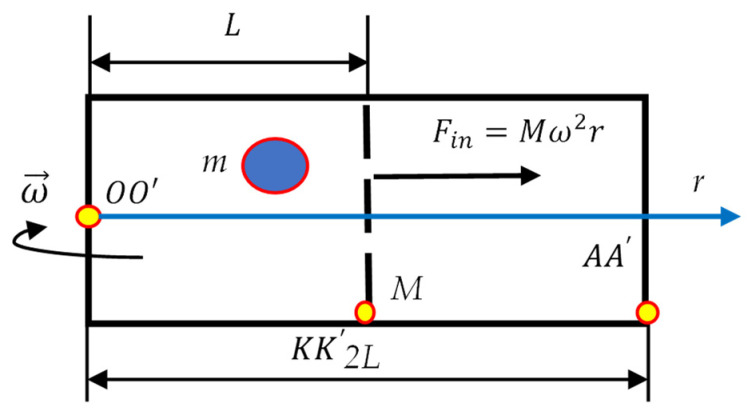
Minimal (Szilard) thermal engine rotated about axis $OO^{\prime }$ with angular velocity $\vec{\omega }$. Particle *m* is initially confined within the left half of the chamber. Partition *M* is free to slip along the chamber. Axes $OO^{\prime },\;\;KK^{\prime },$ and $AA^{\prime }\;\;\mathrm{are}$ perpendicular to the plane of the drawing. Finding the particle m in the left half of the rotating chamber corresponds to the re-cording of 1 bit of information. Displacement of the partition *M* by the inertia force $F_{in}=m\omega^{2}r$ makes the location of particle uncertain, thus corresponding to the erasure of 1 bit of information.

Now address the minimal rotating thermal engine [22,23,24,25,26], built of the chamber, movable partition *M*, which is free to slip in a frictionless way along the chamber, and the particle *m* confined within the chamber as depicted in Figure 4. The motion of the particle is random; in other words, the motion of the particle is thermalized (in other words randomized, for the details see [18]). The entire system may be rotated along the axis $OO^{\prime }\;$(see Figure 4). The single-particle (minimal) thermal engine was introduced by Leo Szilard in 1929 in [22] and implemented and developed recently in [23,24,25,26]. Minimal thermal engine is often used for the demonstrating the Landauer Principle [14,15,16,17,18,27]. We also apply the rotating minimal (Szilard) thermal engine shown in Figure 4 for the clarification of the Landauer Principle. For this purpose, assume that finding the particle *m* in the left half of the rotating chamber corresponds to the recording of 1 bit of information. When the partition is displaced, the location of the particle is uncertain, and this corresponds to the erasure of 1 bit of information. Location of a particle *m* on the left half of the chamber corresponds to “1”, and the uncertain location of the particle corresponds to “0”; thus, the single-particle-based computer provides the binary logical system.

Consider the isothermal expansion of our minimal single-particle gas emerging from the rotation of the minimal thermal engine around axis $OO^{\prime }$, perpendicular to the plane of the drawing, shown in Figure 4. Let us rotate the entire engine with the angular velocity $\vec{\omega }\left(r\right)$, where *r* denotes the current location of the partition (see Figure 4). The inertia force $F_{in}\left(r\right)=M\omega {\left(r\right)}^{2}r$ will displace the partition to the right, thus erasing one bit of information. Consider the Gedanken Experiment isothermal expansion of our single-particle gas. If the expansion due to the rotation is assumed to be isothermal, PV=const holds. This yields Equation (6):

(6) $$\frac{M\omega {\left(r\right)}^{2}r}{S}Sr=const$$

(7) $$\omega {\left(r\right)}^{2}r^{2}=\omega_{0}^{2}L^{2},$$

where *S* and *L* denote the area of the cross-section and initial position of the partition (see Figure 4), $\omega_{0}$ is the initial angular velocity of the partition (chamber). Thus, “isothermal expansion of the minimal gas” will take place when Equation (8) holds:

(8) $$\omega \left(r\right)=\omega_{0}\frac{L}{r}$$

Work performed by the inertia centrifugal force under this expansion is given by Equation (9):

(9) $$A=\int_{0}^{V}P\left(V\right)dV=k_{B}Tln2$$

which exactly coincides with the Landauer bound when the partition divides the volume of the chamber by half [14,15,16,17,18]. This result is quite expectable; we already demonstrated in our recent paper that the inertia forces may be used for erasing information within the minimal thermal engine, which is displaced by translation.

What is, perhaps, less expectable is the fact that the same engine could not be used for recording the information and could not be made reversible. Indeed, when the compression stage of the process is accomplished and 1 bit of information is already erased, let us change the rotation of the minimal thermal engine, shown in Figure 4, from $\vec{\omega }$ to $-\vec{\omega }$; in other words, let us change the clockwise rotation of the chamber to the counter-clockwise. Obviously, the “gas” will not be compressed, and 1 bit of information will not be recorded by the system. Again, exactly as in Section 2.1, in order to compress the gas and consequently to record 1 bit of information, we have to involve the another axis of rotation (for example, axis $AA^{\prime }$ shown in Figure 4). This situation somewhat surprisingly resembles the performance of the cyclic thermal engine, where we need the “hot bath” and “the cold bath” in order to accomplish the closed operation cycle. Similarly, in our rotating thermal engine, we need two axes in order to close the loop. Thus, the rotating single-axis rotating minimal thermal engine enables erasing of information and does not allow recording, perhaps hinting in such a way to the origin of the “arrow of time”. Again, rotation of the minimal thermal engine around axis $KK^{\prime }$ coinciding with the symmetry axis of the chamber/partition system does not enable the displacement of the partition and erasing of information under rotation of the minimal thermal engine.

## 3. Conclusions

It is well-accepted that the statistical approach and thermodynamic laws emerging from this approach are applicable for the systems containing the large number of particles, which is comparable with the Avogadro number [28,29]. However, in the last decades, the thermodynamics of small systems has attracted the attention of investigators [30,31]. The minimal possible thermodynamic device is built from the single-particle system, in which the motion of the particle is supposed to the be “thermalized” (randomized) [23]. Such a minimal thermodynamic system is exemplified by the Szilard thermal engine exploiting the motion of the single particle [22]. The Carnot cycle of such an engine yields the efficiency, which coincides exactly with the efficiency of the “usual” thermal engine [23]. We considered two minimal thermodynamic systems, namely the rotating double-well system comprising the particle and the rotating Szilard thermal engine, shown in Figure 1, Figure 3, and Figure 4 correspondingly. The aforementioned minimal thermal systems were successfully used for the demonstrating of the Landauer Principle, establishing the minimal energy cost necessary for the erasing of information [14,15,16,17,18,32,33]. In both of the suggested systems the inertia (centrifugal) force is used for the recording of the recording/erasing of information. It is demonstrated that the centrifugal force enables recording of information in the rotating double-well system and erasing of information in the rotating system built of the Szilard minimal thermal engine. The Stokes-like friction force acting on the particle destabilizes its equilibrium and promotes recording of information [19,20,21]. The curious feature of the rotating single-axis minimal thermal systems should be emphasized. The aforementioned recording/erasing of information in the rotating single-axis systems is irreversible. The change of the direction of rotation does not enable to reverse the process of recording/erasing of information, thus hinting at the emerging “arrow of time” in these systems. The reversibility of the process of recording/erasing of information is provided by the second axis of rotation, which does not coincide with the initial one. The situation resembles the operation of the thermal engines: the reversibility of the closed loop engines is necessarily provided by two kinds of baths, namely “hot” and “cold” ones. The twin-axes based rotating system will enable development of the minimal rotating Carnot engine, including recording/erasing of information operation stages (the kinetic considerations, related to the “adiabatic” stage of the cycle, are expected to be important for such a Carnot engine [34]). The rotating potential comb enabling recording of information is introduced. Breaking the symmetry of the rotating system makes the recording/erasing of information possible. Thus, the non-obvious interrelation between the initial spatial symmetry of the rotating system and possibility of recording/erasing information within the rotating device is elucidated.
